# Supplementary material for: Oncogenic Role of Secreted Engrailed Homeobox 2 (EN2) in Prostate Cancer
Source: J Clin Med. 2019 Sep 6;8(9):1400. doi: 10.3390/jcm8091400 (PMC6780828; doi:10.3390/jcm8091400)
Supplement: Supplementary file 1 [file jcm-08-01400-s001.pdf]

Oncogenic role of secreted engrailed homeobox 2 (EN2) in prostate cancer

Authors: Gómez-Gómez E[1,2,3,4], Jiménez-Vacas JM[1,2,3,5], Pedraza-Arévalo S[1,2,3,5], López-López F[1,2,3,5], Herrero-Aguayo V[1,2,3,5], Hormaechea-Agulla D[1,2,3,5], Valero-Rosa J[1,3,4], Ibáñez-Costa A[1,2,3,5], León-González AJ[1,2,3], Sánchez-Sánchez R[1,3,6], González-Serrano T[1,3,6], Requena-Tapia MJ[1,3,4], Castaño JP[1,2,3,5], Carrasco-Valiente J[1,3,4], Gahete MD[1,2,3,5] Luque RM[1,2,3,5*].

Affiliations: [1]Maimonides Institute for Biomedical Research of Cordoba (IMIBIC), Córdoba, Spain. [2]Department of Cell Biology, Physiology, and Immunology, Universidad de Córdoba, Córdoba, Spain. [3]Reina Sofia University Hospital, Córdoba, Spain. [4]Urology Service, Reina Sofia University Hospital, Córdoba, Spain. [5]CIBER Fisiopatología de la Obesidad y Nutrición (CIBERobn), Córdoba, Spain. [6]Anatomical Pathology Service, Reina Sofia University Hospital, Córdoba, Spain

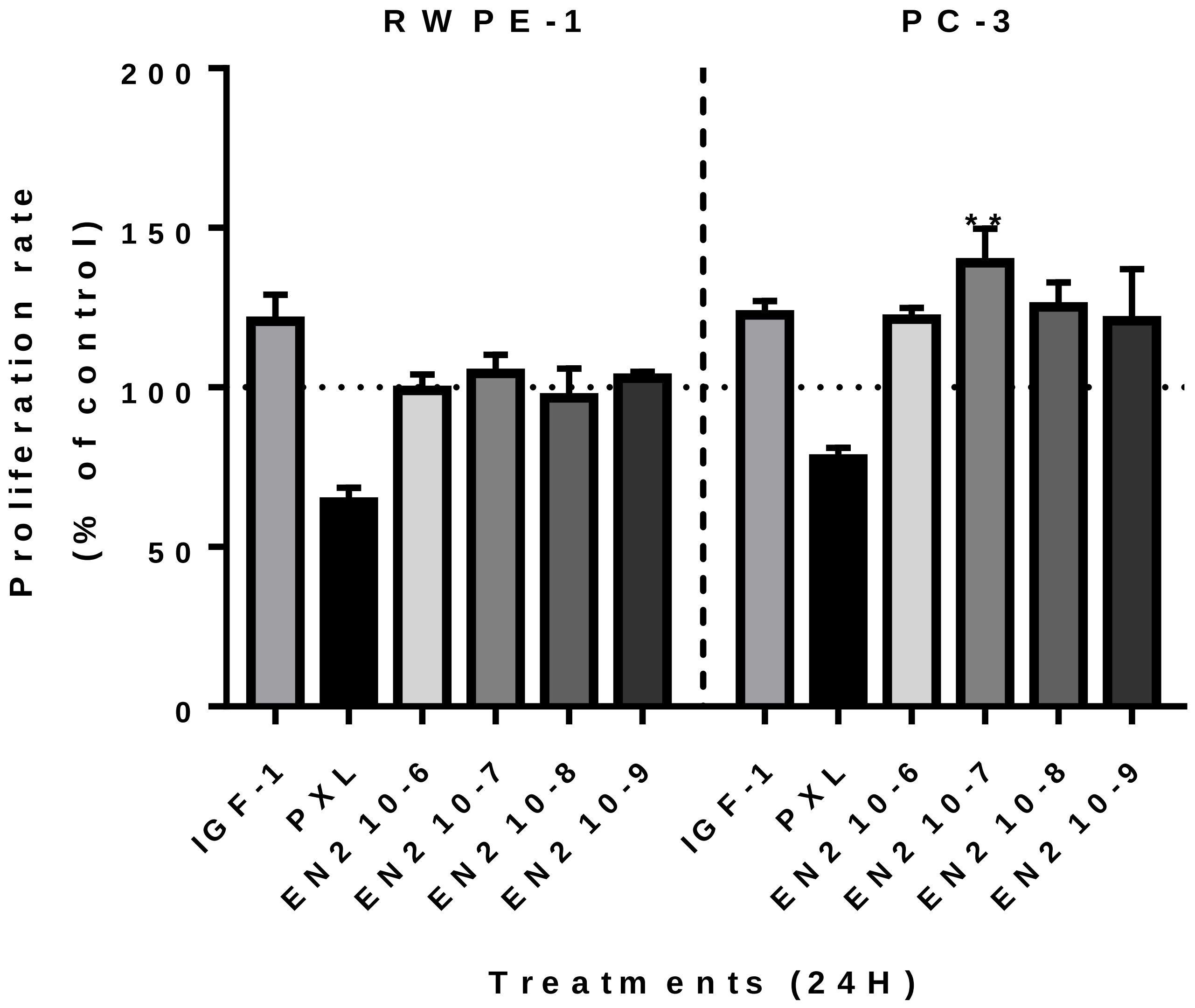

Supplemental figure 1. Dose-response analysis of EN2 treatment in RWPE-1 and PC3 cell proliferation. Proliferation rate in response to EN2 treatment at 10-6 to 10-9 M at 24 h. Data represent mean ± SEM. *, $p \leq 0.05$; **, $p < 0.01$, indicate values that significantly differ from the reference. PXL means paclitaxel

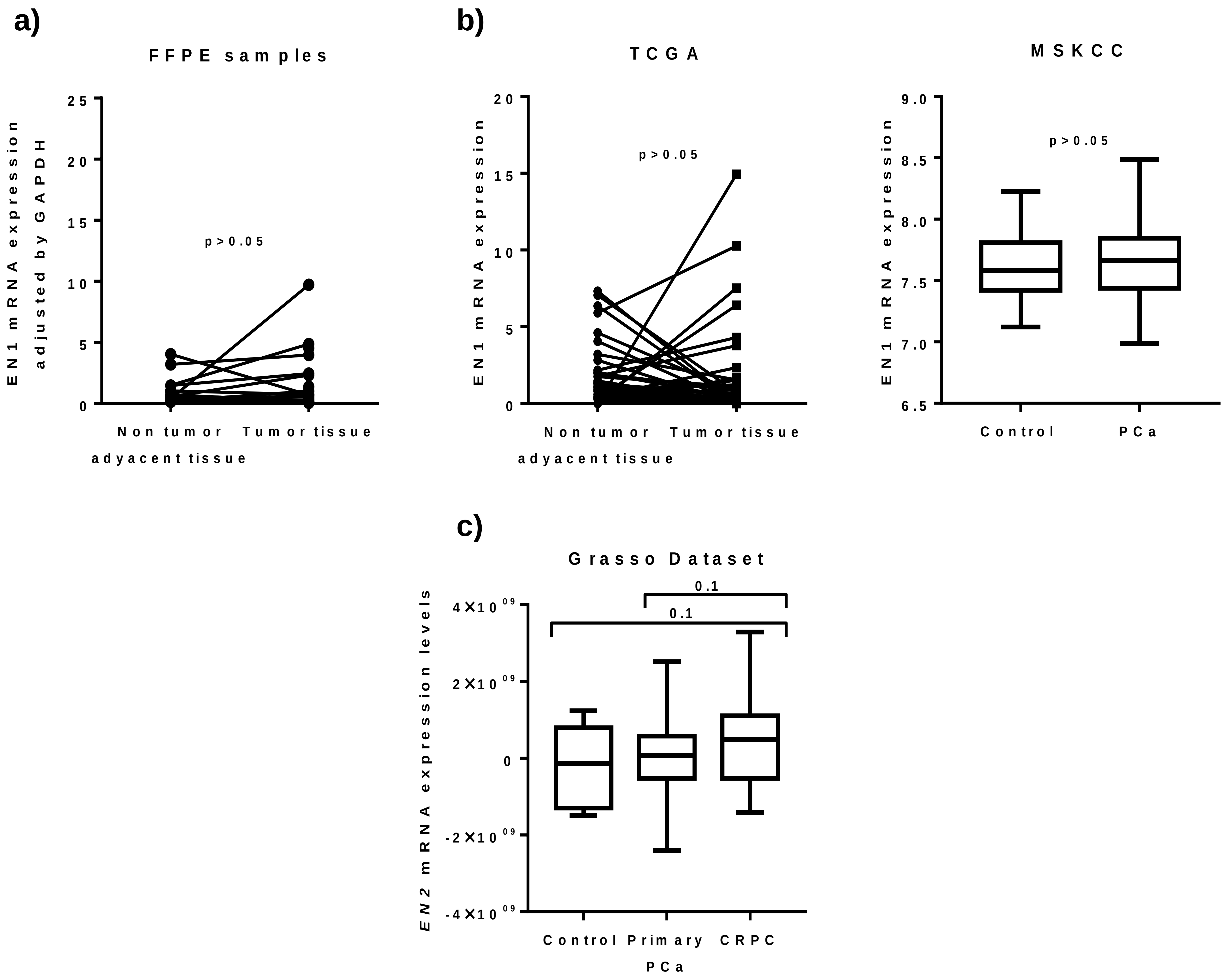

Supplemental Figure 2. Expression of *EN1* in PCa and EN2 in CRPC PCa. **A**. Paired analysis of *EN1* mRNA expression levels in PCa tissue and matched with adjacent non-tumor tissue from 18 FFPE prostatectomy samples. Absolute mRNA levels were determined by qPCR and adjusted by GAPDH housekeeping gene **B.** Analysis of *EN1* mRNA expression levels in 52 PCa tissue and 52 non-tumor adjacent tissue from TGCA data set (left) and analysis of *EN1* mRNA expression levels in 29 non-tumor tissue and 150 PCa tissue from the MSKCC dataset (right). **C.** Analysis of EN2 mRNA expression levels in 12 Control, 49 primary PCa and 27 CRPC samples from the Grasso cohort dataset Data represent mean ± SEM.

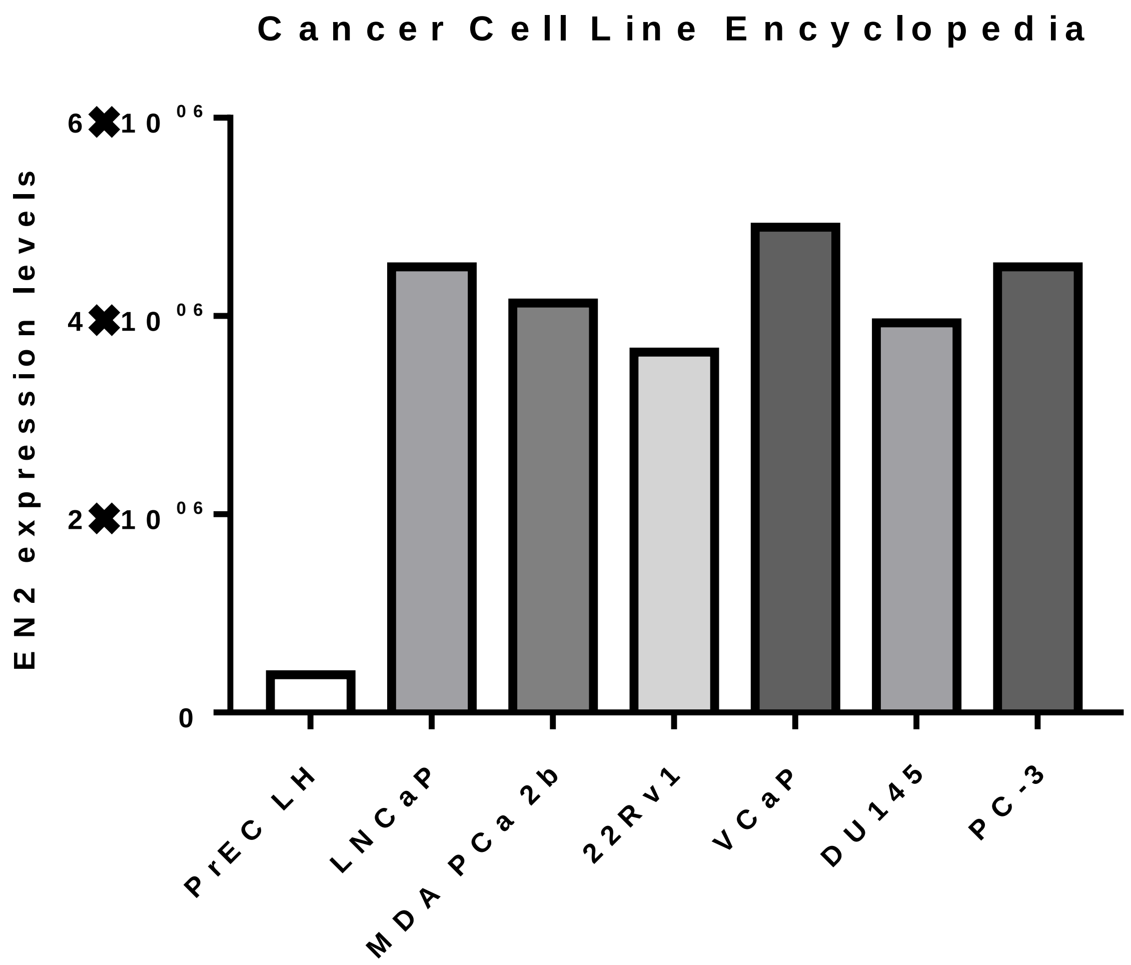

Supplemental figure 3. Free available expression levels of *EN2* in normal and tumoral prostate cell lines, from Cancer Cell Line Encyclopedia (https://portals.broadinstitute.org/ccle).

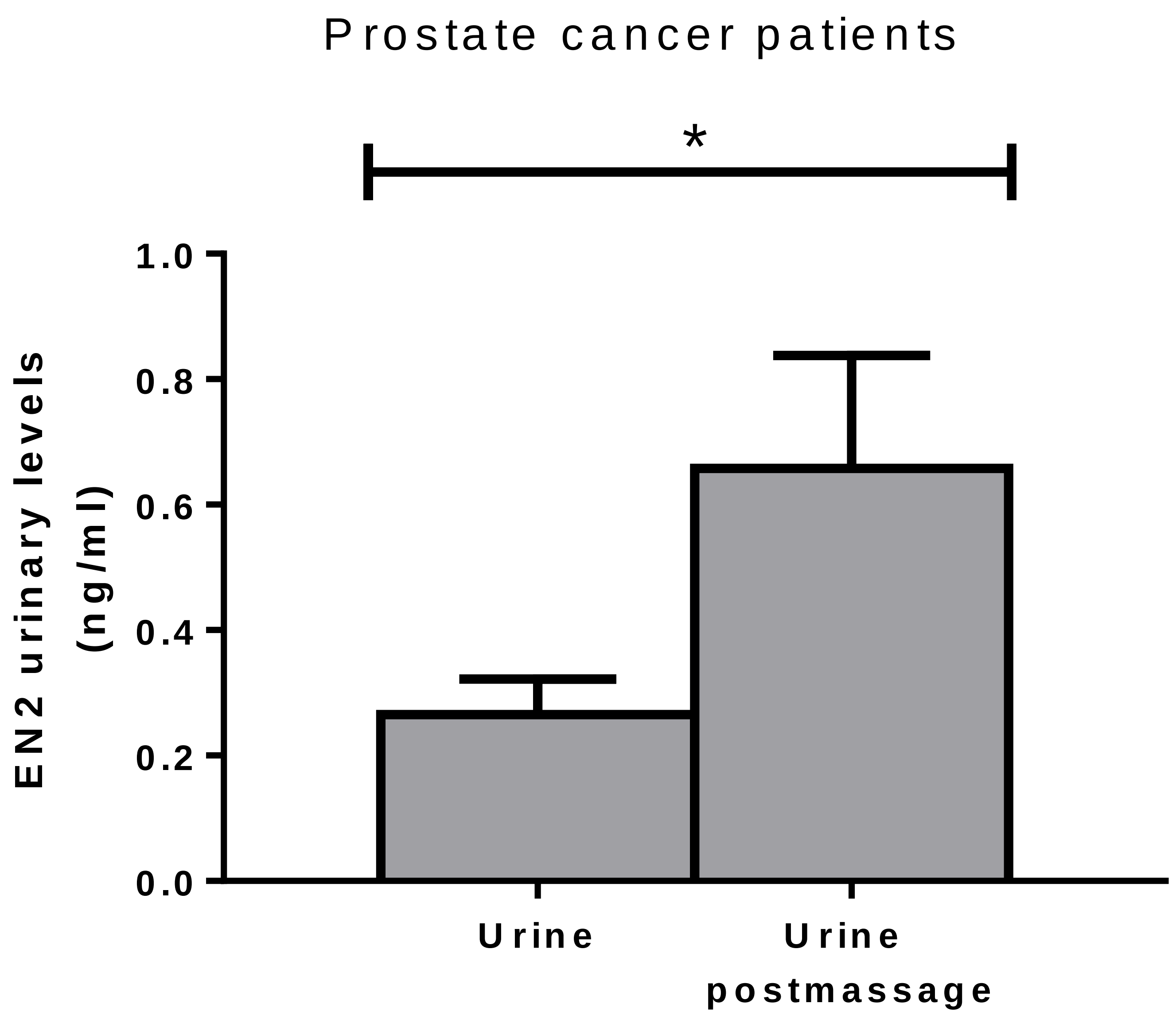

Supplemental figure 4. Urinary levels of EN2 before and after digital rectal examination in the cohort of patients with PCa (n= 24). Data represent mean ± SEM. *, p≤0.05.

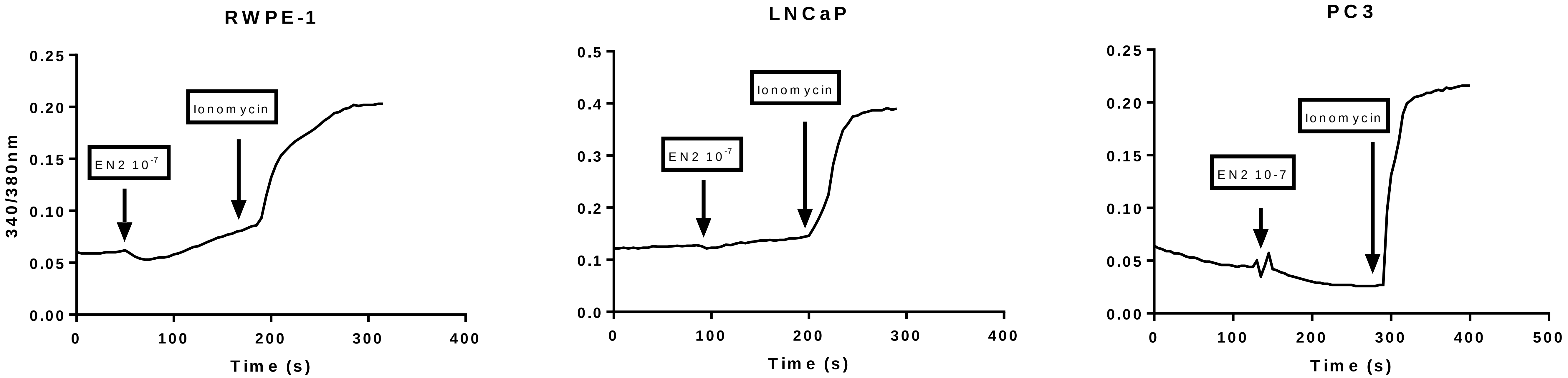

Supplemental figure 5. Representative profiles of $[Ca^{+2}]_i$ in RWPE-1, LNCaP and PC3 cell lines (n=2) in response to EN2 protein.

No changes was observed in response to EN2, while ionomycin elicited the expected response.

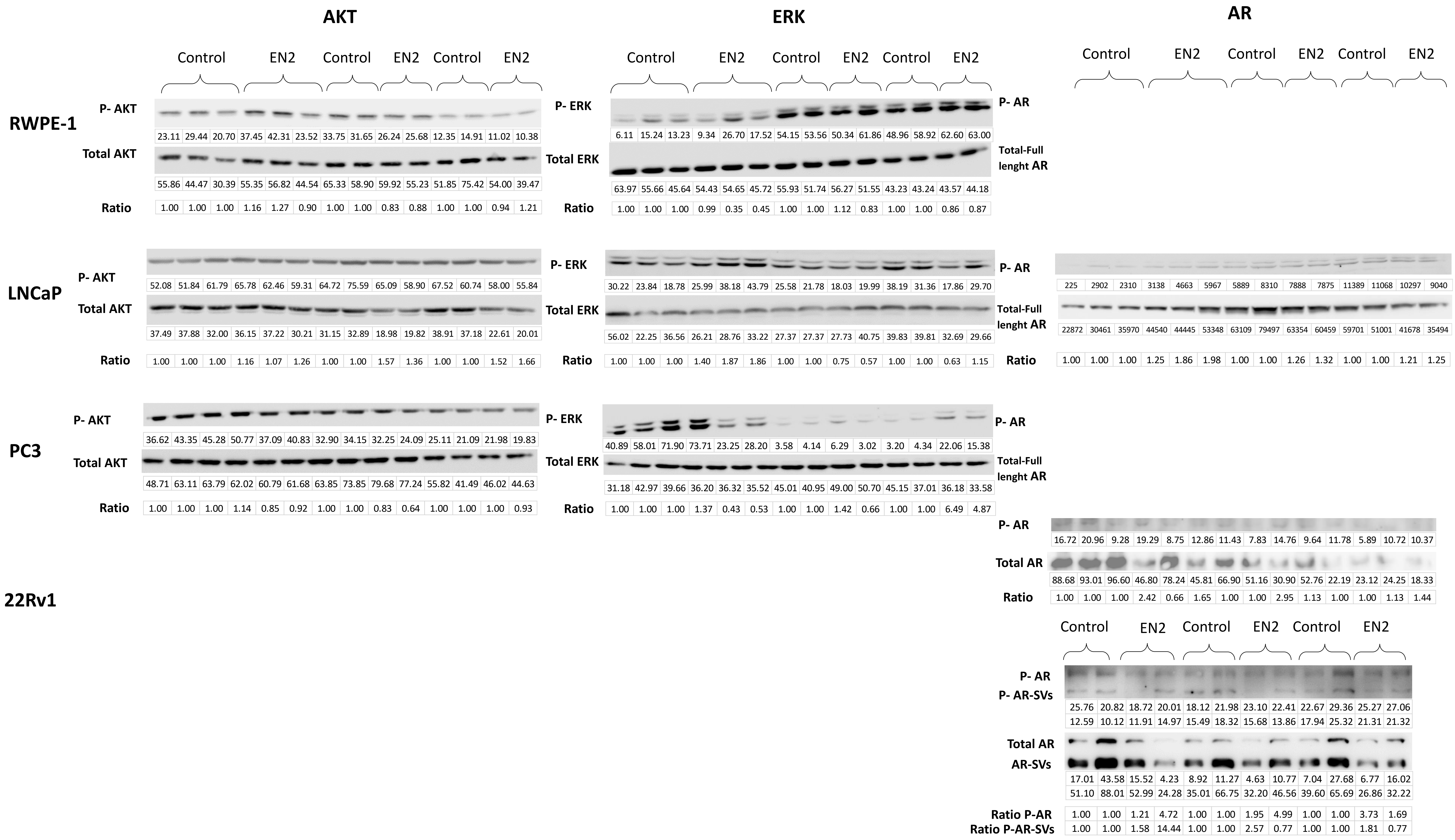

Supplemental figure 6. Western blot images: full-length gels and blots. Phosphorylation of key signalling pathways (AKT, ERK and AR; from left to right) after EN2 treatment during 8 min, compared with non-treated control. Total protein was used as normalizing factor of the respective phosphorylated protein. AR-SVs; AR splice variants. It should to be noted that protein ratios of EN2 treated cells has been compared against protein ratios of control cells (which has been set at 100 %), therefore minimizing the variability of the data. Arbitrary units of densitometry analyses as well as final data (expressed as ratio of control cells) are shown under each blot. * Second western-blot showing not only full-length but also AR-SVs.

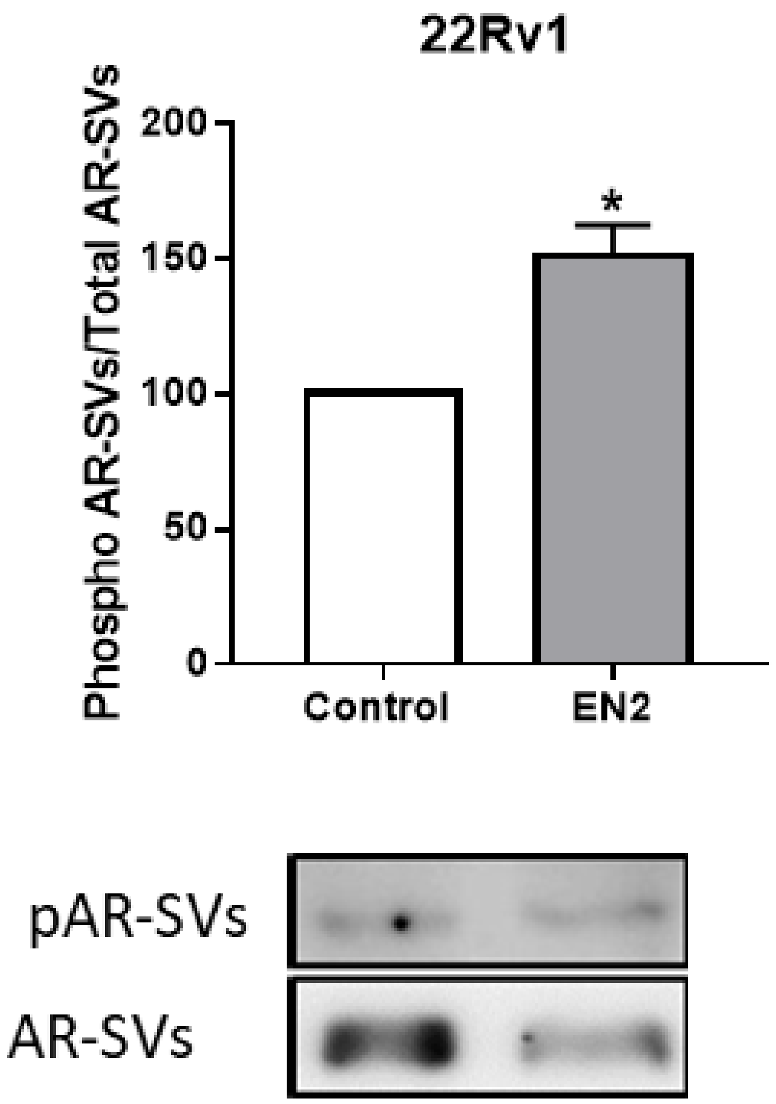

Supplemental figure 7. Phosphorylation of AR splice variants after EN2 treatment in 22Rv1 cells. A. Phosphorylation after EN2 treatment during 8 min, compared with non-treated control (full western blot images in supplemental figure 6). AR-SVs= AR splice variants. Data represent mean ± SEM and they are expressed as percentage of the ratio (set at 100%). *, p ≤0.05; indicate significant differences compared to control.
